# The Idea Is Good, but…: Failure to Replicate Associations of Oxytocinergic Polymorphisms with Face-Inversion in the N170

**DOI:** 10.1371/journal.pone.0151991

**Published:** 2016-03-25

**Authors:** Aisha J. L. Munk, Andrea Hermann, Jasmin El Shazly, Phillip Grant, Jürgen Hennig

**Affiliations:** 1 Justus Liebig University Giessen, Department of Psychology, Personality and Biological Psychology, Otto-Behaghel-Strasse 10F, 35394 Giessen, Germany; 2 Justus Liebig University Giessen, Department of Psychology, Psychotherapy and Systems Neuroscience, Otto-Behaghel-Strasse 10F, 35394 Giessen, Germany; 3 Justus Liebig University Giessen, Faculty of Medicine, Center for Psychiatry and Psychotherapy, Am Steg 28, 35392 Giessen, Germany; University of Toyama, JAPAN

## Abstract

**Background:**

In event-related potentials, the N170 manifests itself especially in reaction to faces. In the healthy population, face-inversion leads to stronger negative amplitudes and prolonged latencies of the N170, effects not being present in patients with autism-spectrum-disorder (ASD). ASD has frequently been associated with differences in oxytocinergic neurotransmission. This ERP-study aimed to investigate the face-inversion effect in association with oxytocinergic candidate genes. It was expected that risk-allele-carriers of the oxytocin-receptor-gene-polymorphism (rs53576) and of CD38 (rs379863) responded similar to upright and inverted faces as persons with ASD. Additionally, reactions to different facial emotional expressions were studied. As there have been difficulties with replications of those molecular genetic association studies, we aimed to replicate our findings in a second study.

**Method:**

Seventy-two male subjects in the first-, and seventy-eight young male subjects in the replication-study conducted a face-inversion-paradigm, while recording EEG. DNA was extracted from buccal cells.

**Results:**

Results revealed stronger N170-amplitudes and longer latencies in reaction to inverted faces in comparison to upright ones. Furthermore, effects of emotion on N170 were evident. Those effects were present in the first and in the second study. Whereas we found molecular-genetic associations of oxytocinergic polymorphisms with the N170 in the first study, we failed to do so in the replication sample.

**Conclusion:**

Results indicate that a deeper theoretical understanding of this research-field is needed, in order to generate possible explanations for these findings. Results, furthermore, support the hypotheses that success of reproducibility is correlated with strength of lower original p-values and larger effect sizes in the original study.

## Introduction

Social interaction and communication is of special importance for human beings. During social interactions faces play an important role, as one can read many social relevant cues from a face. By perceiving a face, humans are very fast and efficient in deciding e.g. about sex, age or ethnicity of the watched person [1]. Furthermore, facial expression is an important social index of potential threat [2]. Identifying and interpreting this information correctly are important skills in order to react appropriately in social interactions [3]. Therefore, expertise in face-perception is one of the best developed functions of the visual system and seems to be mediated by distinct processes in the brain which respond especially to face-stimuli [4]. Molecular-genetic correlates underlying individual differences in face-perception- and processing are of special interest in relation to the science of prosocial behavior. The oxytocinergic system came more and more into the focus of attention in studying differences in prosocial behavior [5]. Different variants of the oxytocin-receptor-gene (OXTR; for a review see [6]), as well as another gene-polymorphism, CD38, which regulates oxytocin-secretion [7–9] play an important role in studying effects of differences in oxytocinergic functioning on individual differences in neurophysiological responsivity, personality and/or behavior. Relationships between differences in functioning of the oxytocinergic system and autism-related symptoms have been postulated, as well as neurophysiological differences in face-processing [5, 6, 9–12].

### N170 and Face-Inversion Effect

One core-component in event-related potentials regarding face-perception- and processing is a negative deflection that occurs between 130-180ms and is largest over posterior-temporal electrodes (N170) [13]. In most electrophysiological studies, the effects of inversion have been found to be specific for faces, as compared to other complex stimuli [14–16], as well as to be stronger in the right-hemisphere [17–20]. Healthy adults exhibit a face-inversion effect (FIE), i.e. show an increase in amplitude and a delay in latency in the N170 in reaction to inverted faces compared to upright ones [4, 14, 21–27]. Through alteration of the familiar configuration of the facial features and spatial relations, inversion disrupts configural face processing, as especially second-order relations, such as the relative size of the features’ spatial relations, are disturbed [28–33]. Therefore, latency and amplitude are sensitive to disruptions in early face processing stages, whereas facial familiarity or recognition does not influence the N170 [14, 34]. The face-inversion effect seen in healthy persons seems to be impaired in people with Autism-spectrum disorder (ASD) [10, 19, 23, 35], as they exhibit longer latencies in reaction to upright faces (compared to controls), but do not exhibit longer latencies in reaction to inverted faces, as controls do. These effects are not evident in reaction to objects. Furthermore, controls do show higher amplitudes in the N170 in the right hemisphere than in the left one, indicating a right-hemispheric specialization in early face processing that is weaker or absent in subjects with ASD [21].

Results regarding the question if emotional facial expression influences the N170 are heterogeneous: while several studies postulate that emotional content of stimuli is processed in later components and not as early as in the N170 [20, 36–41], other studies suggest that affect is associated with a stronger negativity in the N170 [2, 3, 38, 42, 43], which would imply early automatic encoding of emotional facial expressions [2]. A recent meta-analysis, however, came to the conclusion that emotional facial expression indeed influences N170-amplitudes [44].

### Molecular genetic associations of face-processing

Whereas meta-analyses regarding twin-studies suggest a sustainable heritability of different ERP-waveforms [45], there have only been a few studies regarding face processing in association with molecular genetics, which will be reported in the following sections.

#### Serotonin (5-HT)

Serotonergic gene-polymorphisms, especially the serotonin transporter gene SLC6A4 with the serotonin-transporter length polymorphic region (5-HTTLPR) have frequently been associated with the responsivity to stimuli with emotional content (for a review see [46]). ERP-studies investigating associations with reactivity to emotional stimuli and 5-HTTLPR found that carriers of at least one short (s-) allele had higher amplitudes in the error-related negativity (ERN) [47]. In an emotional picture-processing task, s-allele carriers of the 5-HTTLPR reacted stronger in early stages of emotional processing, but not in late ones [48]. Battaglia and colleagues [49] reported associations of s-allele carriers and differences in reaction to emotional faces in the N400, but not in the N170. Similar results were reported from Labuschagne et al. [50] in a citalopram-challenge: Whereas an increase of serotonin led to an enhanced N250 as well as larger amplitudes in the late positive potential (LPP) in reaction to happy (vs. neutral) faces, no effect on the N170 was evident. Schmidt and colleagues [51] reported that the glutamatergic NMDA receptor antagonist ketamine, as well as the 5-HT agonist psilocybin led to reduced N170-amplitudes in reaction to fearful faces. Furthermore, ketamine also led to reduced N170-amplitudes in response to happy faces, whereas this was not the case under influence of psilocybin. As far as we know, no study investigated associations of serotonergic functionality with the FIE, yet.

#### Dopamine (DA)

Regarding dopaminergic neurotransmission, associations of the catechol-O-methyltransferase (COMT) val158met polymorphism and time of onset of the N170 have been reported; hereby, the met-allele was associated with significantly reduced N170-latencies in children [52]. However, most studies that investigate dopaminergic polymorphisms in association with ERPs, report associations with the ERN, the conflict-N400 or the LPP [53–57]. Results regarding face-inversion in association with dopaminergic neurotransmission have not yet been published.

#### Oxytocin (OX)

As reported above, results regarding face-processing and associations with dopaminergic, or serotonergic functioning have been sparse. Therefore, the most promising influencing factor on socially relevant cues, such as faces, might be oxytocinergic neurotransmission, because of various results regarding the influence of oxytocin on social behavior, trust, emotion- and face-recognition, as well as face processing [58]. Waller and colleagues [59] reported influences of oxytocin (intranasal administration) on face-processing of pictures of children’s faces in the N250 and N300 in fathers: view of father’s own child (in comparison to pictures of familiar or unfamiliar children) led to enhanced negativities in the N250 and N300, only in the placebo-condition, with this difference being absent after oxytocin-administration. Most promising were the results from Huffmeijer et al [60], where oxytocin-administration led to increases of amplitudes in reaction to emotional faces in the vertex positive potential (VPP), reflecting the same processes as the N170 [61]. The authors also found that the LPP showed higher amplitudes in general after oxytocin-administration in comparison to the control-group [60]. Therefore, individual differences in oxytocin-secretion-/ and functioning seem to be worthwhile to study in association with processing of faces.

### Oxytocinergic gene polymorphisms

#### Oxytocin-receptor-gene

Different genetic variants of the oxytocin-receptor-gene have been associated with impairments in social behavior [5, 62]. There is only one receptor for Oxytocin: OXTR [62]. OXTR has also been found in the amygdala, which points to the potential modulation of amygdala-activity through OX [63]. One single-nucleotide-polymorphism (SNP), found on intron 3 is SNP rs53576 (G>A), seems to have an inhibiting influence on transcription of the OXTR gene [64]. Its function is, however, not totally clear, yet [6], but it seems to have an influence on social behavior and interactions [6, 65]. In a sample with healthy students, subjects homozygous for the G-allele showed more dispositional and behavioral empathy, measured with the “Reading the Mind in the Eyes test”, than students with at least one A-allele. Furthermore, associations of optimistic behavior [66], and of prosocial temperament and the GG-genotype in comparison to A-allele carriers have been reported. Besides, a significant reduction in amygdala-activity in reaction to negative emotional faces [67], as well as more positive emotions in G-allele-carriers [68] are being postulated. In a sample of 195 Chinese families, an association of SNP rs5357 G-allele-carriers and autism has been reported [69]. This effect could, however, not be replicated in a Caucasian sample [70].

#### CD38

Beside the OXTR-gene, CD38, which is known as a transmembrane glycoprotein with fundamental importance in neuropeptide release [7], is involved in the regulation of the oxytocinergic system as well [7, 71, 72]. Within the CD38-gene, various SNPs can be distinguished. Munesue and colleagues [73] reported that the SNP rs3796863 (C>A), located on intron seven, showed associations with high functioning autism (IQ > 70) in a Caucasian population (US), but not in a Japanese sample. Furthermore, Lerer and colleagues [74] reported associations between several SNPs across the CD38 gene and low functioning ASD, as well as a reduction in CD38 expression in lymphocytes in persons with ASD. Sauer and colleagues [75] showed associations between this SNP (rs3796863) and neural processing of social stimuli, with homozygous C-allele carriers expressing higher activation in the left fusiform gyrus (FG), as well as slower reaction times during processing of neutral and emotional faces. Intranasal application of OX led to reduction of reaction times in CC-homozygous subjects and, therefore, to improved processing of social relevant stimuli [75]. Results regarding genetic variants of the CD38 gene hint to an involvement in the regulation of social interactions and might have important implications for psychiatric disorders, which are characterized by social deficits [76]. Therefore, both polymorphisms, CD38 and OXTR seem to be worthwhile to be studied in associations with individual differences regarding the reaction to social stimuli—such as (emotional) faces.

### Research question

The goal of this study was to find out, if differences in oxytocin-related polymorphisms- namely oxytocin-receptor-gene-polymorphism (rs53576) and CD38-polymorphism (rs3796863) were influencing neurophysiological face-processing in healthy adults. In comparison to results found in association with subjects with ASD and the FIE, we conducted an EEG-study with a face-inversion-paradigm that is similar to the one used by McPartland et al. [10], in a sample of 72 healthy young men. Important questions in this context are, if alterations of the N170 in response to faces and objects are associated with genetic determinants of the oxytocinergic system, and, if these can be observed in healthy subjects. This would point to an influence of genetically determined oxytocinergic gene variants on processing of socially and emotionally relevant cues.

Due to the ongoing discussion about reproducibility problems in psychological- and especially neuroscience [77–79], and in order to see if found effects were stable and reliable, we conducted a replication of the original study with the same paradigm and a comparable sample of 78 healthy young males.

Thus, it was hypothesized:

1. Neutral upright vs. neutral inverted faces (face-inversion) and upright vs. inverted chairs (object-inversion as control stimulus):
  - Stronger amplitudes and longer latencies in reaction to inverted than to upright neutral faces in general are expected.
  - N170-amplitudes and latencies in reaction to faces are expected to be higher and faster than to chairs, respectively.
  - Furthermore, it is expected that risk-allele carriers of OXTR and/or CD38 will not show a FIE, and, therefore, exhibit no shorter N170-latencies and smaller N170-amplitudes in reaction to inverted faces, as well as no lateralization in the right hemisphere. Latencies in reaction to objects (chairs) are expected to be shorter in risk-allele carriers than in non-risk-allele-carriers.
2. Exploratory analyzes would further reveal amplitude differences in reaction to emotional (happy, angry, neutral) facial expressions, with and without associations with OXTR-SNP and/or CD38-genotypes.

## Method

### Participants

#### Study 1

Seventy-two native German-speaking Caucasian healthy young males participated in this study. Subjects had normal or corrected-to-normal vision and were right-handed. EEG-testing was only one part of the study; fMRI-testing and a learning-paradigm took place on different days after EEG-testing. All subjects gave their written informed consent before testing and received a monetary compensation of €10/h for their participation in the entire project. Before testing, participants provided genetic samples with buccal cells from saliva. Five participants had to be excluded from the EEG- and further analyses, as they had more than two standard deviations less trials than average trial number; leaving a final sample of sixty-seven male subjects between 18 and 33 years of age (M = 24.8; SD = 3.04).

Subjects were grouped on genotype-level in AA and AG vs. GG (A+ vs. GG) for rs53576 (OXTR SNP), whereas the risk-allele group is the one with at least one A-allele (A+); as well as grouping on genotype-level for rs3796863 (CD38 SNP) in CC vs. AA and AC, whereas the critical genotype-variant is CC in this case.

#### Study 2

Seventy-seven native German-speaking Caucasian healthy young males participated in this study. Subjects had normal or corrected-to-normal vision and were right-handed. EEG-recording consisted of the face-inversion paradigm and an emotional Stroop task, which was balanced, so that 50% of the subjects started emotional Stroop task and the other 50% started with the face-inversion paradigm. All subjects gave their written informed consent before testing and received a monetary compensation for their participation in the project. Before testing, participants provided genetic samples with buccal cells from saliva. Five participants had to be excluded from the EEG-analyzes, as they had more than two standard deviations less trials than average-trial number, leaving a final sample of seventy-two male subjects between 18 and 32 years of age (M = 24.30; SD = 3.20).

The face-inversion paradigm, ERP-recordings- and analyzes, genotyping, and statistical analyzes were conducted the same way as in study 1.

Both studies were approved by the ethics committee of the faculty of psychology of the Justus-Liebig-University Giessen and comply with the Declaration of Helsinki.

### Face-Inversion paradigm

Stimuli consisted of gray-scale digital images of neutral, happy and angry faces, 20 per category, 10 women and 10 men—upright and inverted (KDEF-database; [80]), as well as of a set of 20 chairs (with kind regards from the Laboratoire de Recherché Experimentale, Institute de Psychologie, Université de Lausanne, CH), upright and inverted. Pictures were standardized for size, contrast and luminosity. Pictures of faces, as well as the pictures of chairs were cut out in an oval form, so that hair did not influence face-perception. Additionally, subjects had to react to a grayscale image of a butterfly (appearing 60 times across all blocks) by pressing a button of a response pad (Cedrus RB-730, Cedrus Corporation, San Pedro, CA, USA), in order to keep attention stable during the task. All images had a resolution of 420x520 pixels. Stimuli were centrally presented on a 15” computer screen with a black background. The task consisted of six blocks with 90 trials each, summing up to 540 trials with randomized presentation of the stimuli. In the overall paradigm, each face and each chair was presented three times upright and three times inverted, leading to 60 trials per category.

Stimuli were presented for 750ms. A central fixation cross was presented during a randomized inter-trial-interval (1000-1500ms; M = 1250ms). There was a break of 20s after every block. Participants were seated in a comfortable chair at 60cm distance to the screen. Stimulus presentation and response recording was conducted with Presentation 16.3 software (Neurobehavioral Systems Inc., Albany, CA, USA) and a Pentium (Intel Corporation, Santa Clara, CA, USA) based personal computer. SPSS Statistics 21 was used for statistical analyses (IBM Corp., Somer, NY, USA).

### ERP recordings and analyzes

EEG signals were amplified using a 0.1–80 Hz bandpass filter (BrainAmpDC amplifier; Brain Products GmbH, Gilching, Germany) and a sampling rate of 250Hz at 32 scalp sites according to the 10-20-system. Active Ag/AgCl electrodes were used (actiCAP 32Ch, Brain Products GmbH, Herrsching, Germany). Impedances were kept below 7kΩ. Vertex electrode was used as online-reference. The data were processed digitally offline, using Brain Vision Analyzer software (Brain Products GmbH, Gilching, Germany). Data were filtered using a 30 Hz low pass filter, manually inspected for artifacts that were not caused by eye movements and, subsequently, excluded. To remove eye-movement artifacts, an Independent Component Analyses (ICA-) based correction algorithm was applied as implemented in the Brain Vision Analyzer. Thereafter, data were re-referenced to an average reference, filtered using a 0.5 Hz high pass- and a 50 Hz notch filter and segmented (stimulus-locked; -200ms to 1000ms). Segments were averaged for each experimental condition and individual; consisting of 54 trials on average (SD = 4.9) in the first study, and 54 trials in the second study (SD = 5.2). For statistical analyzes of the N170, amplitudes and latencies in the time window between 120 and 220ms were extracted at temporo-parietal-occipital electrodes P7, P8, PO9, PO10, TP9 and TP10. Electrode choice was based on visual inspection of the Grand Average waves and in accordance with the typical topography of the N170.

### Genotyping

DNA was extracted from buccal cells and purification of genomic DNA was performed with a standard commercial extraction kit (MagNA Pure LC DNA Isolation Kit I; Roche Diagnostics, Mannheim, Germany) in a MagNA Pure LC System (Roche, Mannheim, Germany). Genotyping of the rs3796863 polymorphism was conducted with a commercial TaqMan^®^ SNP Genotyping Assay (C___1216944_10, Applied Biosystems, Carlsbad, USA) on a Mastercycler^®^ ep realplex (Eppendorf, Hamburg, Germany) according to manufacturers’ standard protocol. Genotyping of SNP rs53576 was conducted using the commercial TaqMan^®^ SNP Genotyping Assay (C___3290335_10, Applied Biosystems, Carlsbad, USA) on a Mastercycler^®^ ep realplex (Eppendorf, Hamburg, Germany) according to manufacturers’ standard protocol.

### Statistical Analyzes

Amplitudes/latencies of the N170 were entered into two separate ANOVAs for repeated measurements:

1. The within-subject factors electrode (P7, P8, PO9, PO10, TP9 and TP10), perspective (upright vs. inverted), category (neutral face vs. chair), hemisphere (left vs. right), and the between-subject factors genotypes (GG vs. A^+^ in OXTR; CC vs. A^+^ in CD38).
2. In the exploratory analyses of emotional effects on N170, ANOVAs for repeated measurements with within-subject factors electrode (P7, P8, PO9, PO10, TP9 and TP10), emotion (happy, angry, and neutral), hemisphere (left vs. right) and the between-subject-factors genotypes (GG vs. A^+^ in rs53576; CC vs. A^+^ in rs3796863) were calculated.

Post-hoc tests were calculated with Bonferroni-correction for multiple comparisons. Greenhouse-Geisser correction was applied in case of violation of sphericity assumption.

## Results

### Distribution of genotypes

#### Study 1: CD38 rs3796863

Thirty-five male subjects had at least one A-allele and were therefore grouped as A^+^; thirty-six were homozygous C-allele-carriers. One subject could not be genotyped for rs3796863 and was therefore excluded from further analyses. Genotype distribution did not deviate from Hardy-Weinberg-Equilibrium (*χ*^2^ = 0.20, *p* = .65).

#### Study 1: OXTRS rs53576

Thirty-six male subjects were homozygous G-allele-carriers; thirty-six had at least one A-allele and therefore grouped as A1^+^. Genotype distribution did not deviate from Hardy-Weinberg-Equilibrium (*χ*^2^ = 3.18, *p* = .07).

#### Study 2: CD38 rs3796863

Thirty-one male subjects had at least one A-allele and were therefore grouped as A^+^; forty-six were homozygous C-allele-carriers. Genotype distribution did not deviate from Hardy-Weinberg-Equilibrium (*χ*^2^ = 0.25, *p* = .61).

#### Study 2: OXTR rs53576

Forty-one male subjects had at least one A-allele and were therefore grouped as A^+^; thirty-six were homozygous G-allele-carriers. Genotype distribution did not deviate from Hardy-Weinberg-Equilibrium (*χ*^2^ = 0.21, *p* = .64).

### General Face-Inversion (Neutral faces—inverted neutral faces & chairs—inverted chairs)

#### Amplitudes Study 1: General effects

Results show a main effect for perspective (upright vs. inverted), with higher negative amplitudes in reaction to inverted images, a main effect of stimulus category with stronger negative amplitudes in reaction to faces than to chairs, as well as stronger amplitudes in the right hemisphere. Perspective and stimulus-category (faces vs. chairs) interacted significantly, with higher negativities in reaction to inverted faces than to inverted chairs. Furthermore, results show an interaction of perspective and hemisphere, with a stronger right-hemispheric lateralization in the inverted position (see Table 1 below).

**Table 1:** Mean amplitude (μVolt) and Standard Error of the Mean (SEM) in response to position of neutral faces (upright vs. inverted) and chairs (upright vs. inverted); Hemisphere (HEM), Oxytocin-receptor-gene-polymorphism rs53576 (OXTR).

	Study 1		Study 2	
	Mean amplitude (μVolt)	SEM	Mean amplitude (μVolt)	SEM
**Face upright**	-3.60	0.30	-4.85	0.33
**Face inverted**	-4.46	0.38	-6.50	0.40
**Chair upright**	-1.63	0.19	-1.29	0.23
**Chair inverted**	-1.51	0.21	-1.12	0.24
**Face upright HEM right**	-3.95	0.39	-5.43	0.39
**Face upright HEM left**	-3.25	0.27	-4.15	0.32
**Face inverted HEM right**	-5.13	0.47	-7.29	0.47
**Face inverted HEM left**	-3.78	0.36	-5.55	0.38
**OXTR GG right hemisphere**	-2.72	0.41	-3.76	0.41
**OXTR A+ right hemisphere**	-3.40	0.45	-3.72	0.43

#### Amplitudes Study 1: Genotype-interactions

There was no main effect for genotype (GT) in both polymorphisms in any condition, but an interaction of hemisphere and OXTR-genotype, with right-hemispheric lateralization in the A-allele-carriers, but not in the GG-group. No additive effects or interactions of OXTR and CD38 were evident.

#### Amplitudes Study 2: General effects

Results show a main effect for perspective (upright vs. inverted), with higher negative amplitudes in reaction to inverted images, a main effect of stimulus category with stronger negative amplitudes in reaction to faces than to chairs, as well as stronger amplitudes in the right hemisphere (Table 1). Perspective and stimulus category (faces vs. chairs) interacted significantly, with higher negativities in reaction to inverted faces than to inverted chairs. The interaction of perspective and hemisphere, with a stronger right-hemispheric lateralization in the inverted perspective, was significant. General effects are comparable to the results in study 1.

#### Amplitudes Study 2: Genotype-interactions

There was no main effect for genotype in both polymorphisms, and no interaction of hemisphere and OXTR-genotype, as it has been found in study 1. Furthermore, no additive effects of OXTR and CD38 were evident. Results of both studies are summarized in Tables 1 and 2.

**Table 2:** Results of ANOVAs with repeated measurements regarding N170-amplitudes in reaction to upright and inverted neutral faces, and objects (chairs); Hemisphere (HEM), perspective (upright vs. inverted picture), Oxytocin-receptor-gene-polymorphism rs53576 (OXTR).

	Study 1			Study 2		
	df: 1, 62			df: 1, 68		
	*F*	*p*	*η*_*p*_^*2*^	*F*	*p*	*η*_*p*_^*2*^
**Face vs. Object**	104.55	**<. 01**	.628	223.23	**< .01**	.767
**Face-inversion**	23.99	**< .01**	.279	43.98	**< .01**	.393
**Face-inversion vs. Object-inversion**	33.82	**< .01**	.353	106.51	**< .01**	.610
**Perspective x HEM**	7.65	**< .01**	.110	6.46	**.011**	.087
**OXTR x HEM**	4.83	**.032**	.072	.644	.425	.009

#### Latencies Study 1: General effects

Concerning latencies, results indicate a main effect for perspective, with longer latencies in reaction to inverted faces (see Table 3 below), as well as a main effect for stimulus category with shorter latencies in reaction to faces than to chairs. There was no main effect of hemisphere, but a stronger right-hemispheric lateralization in reaction to inverted images.

**Table 3:** Mean latencies (ms) and standard error of the mean (SEM) in response to perspective of neutral faces (upright vs. inverted) and chairs (upright vs. inverted); Hemisphere (HEM).

	Study 1		Study 2	
	Mean latency (ms)	SEM	Mean latency (ms)	SEM
**Face upright**	170.18	1.67	171.11	1.32
**Face inverted**	174.15	1.48	176.63	1.18
**Chair upright**	176.41	2.32	184.20	1.93
**Chair inverted**	182.30	2.11	189.51	1.77
**Face upright HEM right**	169.25	2.02	170.08	1.41
**Face upright HEM left**	171.11	2.20	172.30	1.53
**Face inverted HEM right**	172.87	1.68	176.70	1.32
**Face inverted HEM left**	175.41	1.89	176.72	1.27

#### Latencies Study 1: Genotype-interactions

**Fig 1:**
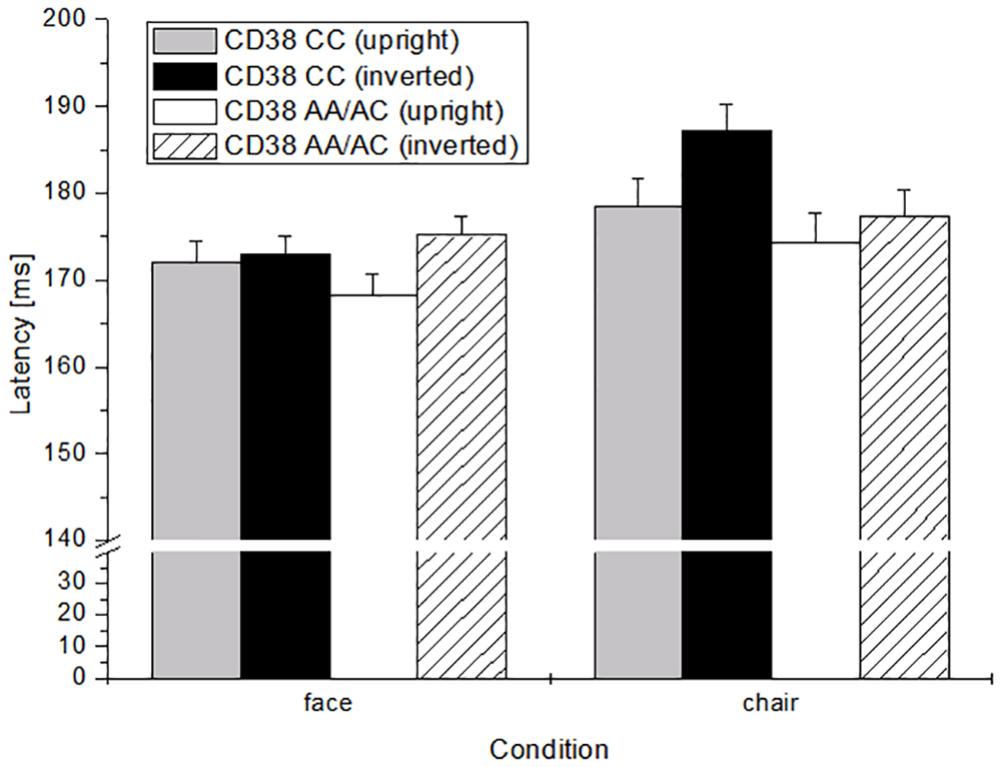
N170-latencies in reaction to upright and inverted faces and chairs in dependence of CD38-allele-frequency. Error bars reflect the standard error of the mean (SEM).

A three-way-interaction of CD38 GT x stimulus category x position was shown, with longer latencies in reaction to inverted chairs in risk-carriers (CC), as well as longer latencies in reaction to inverted faces in the non-risk-carriers (A+) (Fig 1). No interaction effects of OXTR or additive/interaction effects of OXTR with CD38 were evident.

#### Latencies Study 2: General effects

Concerning latencies, results indicate a main effect for perspective, with longer latencies in reaction to inverted faces (see Table 3), as well as a main effect for stimulus category with longer latencies in reaction to faces than to chairs. There was no main effect of hemisphere, and no right-hemispheric lateralization in reaction to inverted images.

#### Latencies Study 2: Genotype-interactions

Neither an effect of genotype, nor an interaction of CD38-genotype, stimulus category and perspective, as in study 1 was evident. Furthermore, no interaction-effects of OXTR or additive effects of OXTR with CD38 could be shown. Results regarding N170-latencies of both studies are summarized in Tables 3 and 4.

**Table 4:** Results of ANOVAs with repeated measurements regarding N170-latencies in reaction to upright and inverted neutral faces, and objects (chairs); Hemisphere (HEM), perspective (upright vs. inverted picture), CD38-SNP rs379863 (CD38); category (faces/chairs).

	Study 1			Study 2		
	df: 1, 62			df: 1, 68		
	*F*	*p*	*η*_*p*_^*2*^	*F*	*p*	*η*_*p*_^*2*^
**Face vs. Object**	14.33	**<. 01**	.188	90.59	**< .01**	.571
**Face-inversion**	37.01	**< .01**	.374	44.31	**< .01**	.395
**Face-inversion vs. Object-inversion**	.697	.407	.011	.029	.866	.000
**Perspective x HEM**	4.59	**.036**	.069	.005	.942	.000
**CD38 x category x perspective**	6.44	**.014**	.094	.895	.348	.013

### Emotional face-inversion (positive, negative and neutral faces)

#### Amplitudes Study 1: General effects

A general FIE was evident, with stronger negative amplitudes in reaction to inverted faces, as well as a main effect of hemisphere with higher negative amplitudes in the right hemisphere. Concerning different facial expressions, amplitudes in reaction to happy faces were significantly higher than towards neutral, but not to angry ones, irrespective if upright or inverted (see Table 5 below). Furthermore, results indicate an interaction of perspective and hemisphere, with higher amplitudes in reaction to inverted faces in the right hemisphere (which was evident in reaction to neutral faces as well).

**Table 5:** Mean difference (μVolt) between facial expressions, standard error of the mean (SEM);

	Study 1			Study 2		
Emotion	Mean difference (μVolt)	SEM	*p*^b^	Mean difference (μVolt)	SEM	*p*^b^
**happy vs. angry**	-.20	0.15	0.55	-.21	.050	.064
**happy vs. neutral**	-.17	0.05	< .01**	-.30	.067	< .01**
**angry vs. neural**	.03	0.14	1	-.08	.076	.763

** The mean difference is significant at the .01 level;

^b^ Adjustment for multiple comparisons: Bonferroni.

#### Amplitudes Study 1: Genotype-Interactions

**Fig 2:**
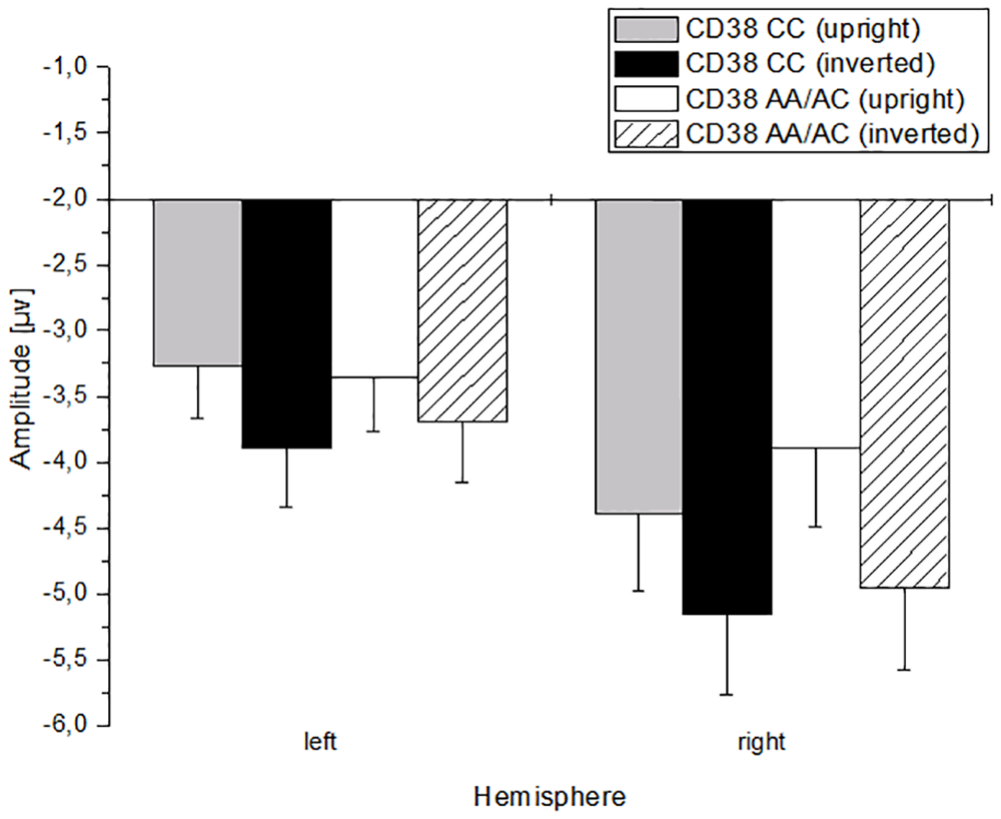
N170-amplitudes in reaction to upright and inverted neutral faces in dependence on allele-frequency of CD38 and hemisphere. Error bars reflect the standard error of the mean (SEM).

An interaction of OXTR with hemisphere indicates that risk-allele-carriers show a stronger lateralization in the right hemisphere—as seen in reaction to neutral faces as well. Furthermore, a three-way-interaction of CD38-GT x perspective x hemisphere indicates that subjects carrying risk-alleles (CC) of the CD38-SNP show a stronger lateralization in the right hemisphere in reaction to upright and inverted faces, whereas the non-risk-group expresses this lateralization only in reaction to inverted faces (Fig 2). No interaction or additive effects of OXTR with CD38 could be shown (see Table 6 below).

**Table 6:** Results of ANOVAs with repeated measurements regarding N170-amplitudes in reaction to upright and inverted happy, and neutral faces; Hemisphere (HEM), perspective (upright vs. inverted picture), CD38-SNP rs379863 (CD38), Oxytocin-receptor-gene-polymorphism rs53576 (OXTR).

	Study 1			Study 2		
	df: 2, 61			df: 2, 67		
	*F*	*p*	*η*_*p*_^*2*^	*F*	*p*	*η*_*p*_^*2*^
**Face-inversion**	54.15	**< .01**	.466	77.21	**< .01**	.532
**Happy vs. Neutral Faces**	5.23	**< .01**	.146	9.80	**< .01**	.226
**Hem**	11.41	**< .01**	.155	18.12	**< .01**	.210
**Face-inversion x HEM**	8.56	**< .01**	.121	4.67	**< .05**	.064
**OXTR x HEM**	7.91	**< .01**	.113	.625	.432	.009
**CD38 x FI x HEM**	4.04	**< .05**	.061	.717	.400	.010

#### Amplitudes Study 2: General effects

Amplitudes were higher in reaction to inverted faces in general. A main effect of hemisphere indicates higher negative amplitudes in the right hemisphere. Concerning different facial expressions, amplitudes in reaction to happy faces were significantly higher than to neutral-, but not to angry ones, irrespective if upright or inverted (Table 5). Furthermore, results indicate an interaction of perspective and hemisphere, with stronger amplitudes in reaction to inverted faces in the right hemisphere. Effects are comparable to those in study 1.

#### Amplitudes Study 2: Genotype-interactions

No interaction of OXTR and hemisphere, and no interaction of CD38-GT, perspective and hemisphere, as in study 1, were evident. Furthermore, interactions or additive effects of OXTR with CD38 could not be found. Results regarding N170-amplitudes in reaction to emotional faces are summarized in tables 5 and 6.

#### Latencies Study 1: General effects

Latency differed significantly in dependence of perspective, with longer latencies in reaction to inverted faces. Latencies were longer in reaction to angry than to happy faces, but not to neutral ones (Table 7). Latencies did not differ in dependence of hemisphere.

**Table 7:** Mean latency difference (ms) between facial expressions and standard error of the mean (SEM);

	Study 1			Study 2		
Emotion	Mean difference (ms)	SEM	Significance^b^	Mean difference (ms)	SEM	Significance^b^
**happy vs. angry**	-3.32	1.10	< .05*	-2.58	0.40	< .01**
**happy vs. neutral**	-1.3	0.54	.06	-1.08	0.32	< .01**
**angry vs. neutral**	2.01	0.97	.127	1.49	0.42	< .01**

* The mean difference is significant at the .05 level;

** The mean difference is significant at the .01 level;

^b^ Adjustment for multiple comparisons: Bonferroni.

#### Latencies Study 1: Genotype-interactions

**Fig 3:**
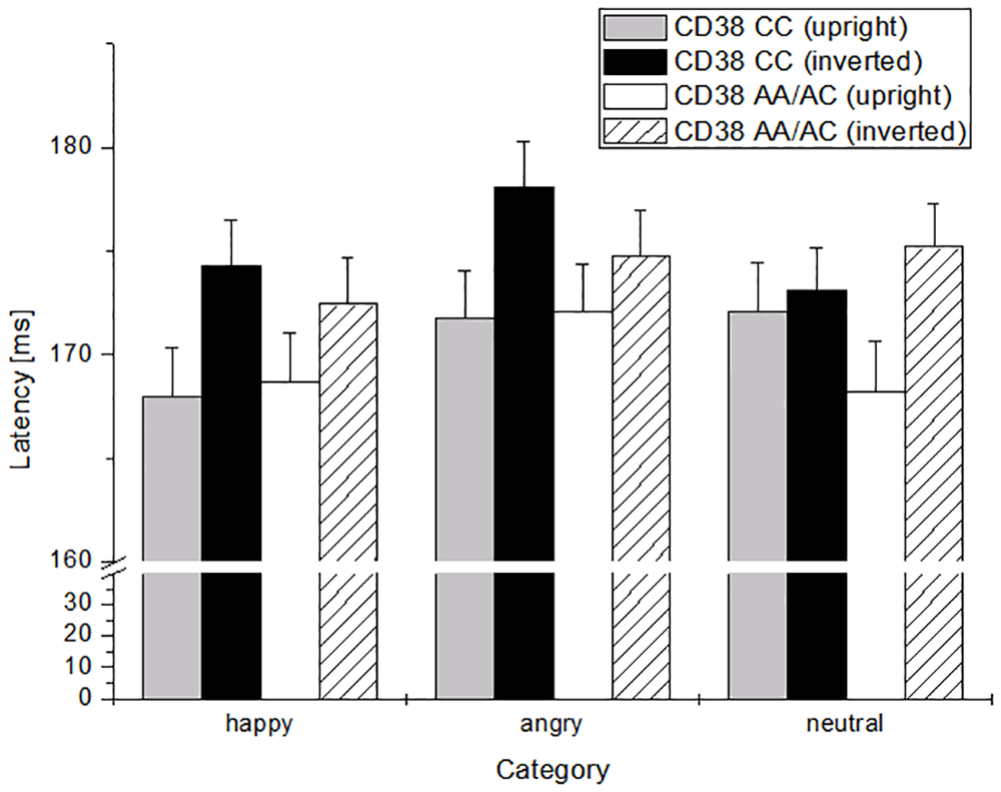
N170-latencies in reaction to happy, angry, and neutral upright and inverted faces in dependence of CD38-allele-frequency. Error bars reflect the standard error of the mean (SEM).

CD38-GT interacted with perspective and facial expression, indicating longer latencies in reaction to angry inverted faces in risk-allele-carriers (CC) than to neutral or happy inverted faces, indicating a present FIE for risk-carriers of CD38, and an absent FIE for CD38-non-risk-carriers (Fig 3). Besides, OXTR-GT interacted significantly with hemisphere and emotion, with shorter latencies in the right hemisphere in reaction to upright angry faces in the risk-allele-group. No interaction or additive effects of OXTR with CD38 were evident.

#### Latencies Study 2: General effects

Latency differed significantly in dependence of perspective, with longer latencies in reaction to inverted faces. Latencies were longer in reaction to angry and to neutral faces compared to happy ones. Latencies did not differ in dependence of hemisphere. Therefore, general results are similar to those of study 1.

#### Latencies Study 2: Genotype-interactions

CD38-GT did not interact with perspective and facial expression, as in study 1. Besides, OXTR-GT did not interact significantly with hemisphere and emotion, as found in study 1. Furthermore, no additive effects of OXTR with CD38 were evident. Results of both studies regarding N170-latency-effects in reaction to emotional faces are summarized in tables 7, 8 and 9.

**Table 8:** Mean latency (ms) and standard error of the mean (SEM) for different facial expressions (happy vs. angry vs. neutral) presented upright and inverted.

	Study 1		Study 2	
Facial expression	Mean latency (ms)	SEM	Mean latency (ms)	SEM
**Happy**	170.86	1.54	169.78	1.32
**Angry**	174.18	1.28	172.21	1.29
**Neutral**	172.16	1.46	171.25	1.35

**Table 9:** Results of ANOVAs with repeated measurements regarding N170-latencies in reaction to upright and inverted happy and neutral faces (emotion); Hemisphere (HEM), perspective (upright vs. inverted picture), CD38-SNP rs379863 (CD38), Oxytocin-receptor-gene-polymorphism rs53576 (OXTR).

	Study 1			Study 2		
	df: 2, 61			df: 2, 67		
	*F*	*p*	*η*_*p*_^*2*^	*F*	*p*	*η*_*p*_^*2*^
**FI**	27.89	**< .01**	.310	89.57	**< .01**	.568
**Angry vs. Happy Faces**	6.82	**< .01**	.099	22.54	**< .01**	.249
**OXTR x HEM x emotion**	3.54	**< .05**	.054	.287	.751	.004
**CD38 x perspective x emotion**	3.53	**< .05**	.054	.737	.481	.011

## Discussion

General effects of face-inversion, with higher amplitudes in reaction to inverted than to upright faces and higher amplitudes in reaction to faces than to chairs (manipulation check) were evident in the first, as well as in the replication study. Furthermore, effects of emotional faces, with higher amplitudes and shorter latencies in reaction to happy than to neutral faces were also present in the first, as well as in the replication study.

Results of the first study indeed indicate associations of different variants of oxytocinergic gene-polymorphisms with amplitude and latency of the N170, with a stronger right-hemispheric lateralization in reaction to faces in OXTR risk-group (A+), an absent FIE in risk-carriers of CD38 (CC), as well as longer latencies in reaction to angry inverted faces in CD38-risk-allele carriers. These genotype-interactions could not be replicated in the second study, though.

### Face-Inversion Effect

Reactions to upright and inverted faces vs. objects (in this case: chairs) were as expected: amplitudes in response to inverted pictures were higher, and amplitudes in reaction to faces were stronger than to chairs. Lateralization showed—as expected—higher negative amplitudes in the right hemisphere, being stronger in reaction to faces than to chairs. Furthermore, genotype of the OXTR-gene polymorphism rs53576 interacted significantly with hemisphere, indicating that the so-called risk-allele-carriers showed—different as expected—a stronger right-hemisphere-lateralization than GG-homozygous. As the other results regarding inversion effects are replicating former findings, results regarding GT-hemisphere-interaction direct in the other direction than expected. The expectation was that risk-allele carriers would react similar as persons with ASD or their relatives, where a lack of lateralization was found—but the opposite was the case: risk-allele carriers showed a stronger lateralization than the so-called non-risk-group, independent of face-inversion or category. Several reasons can account for this effect: results regarding SNP rs53576 have been heterogeneous, with differences in ethnic groups such as Europeans and Asians, so that the “risk-allele” varied in dependence of sample and/or ethnicity [6, 62].

General reactions in latency of N170 were as expected, with longer latencies to inverted pictures and longer latencies in response to chairs than to faces. Furthermore, the CD38-risk group showed longer latencies in reaction to inverted chairs than to faces. This is an effect which was postulated—as subjects with ASD show the same reaction-pattern. It might indicate that attention to objects is more evident in risk-allele-carriers of this CD38-SNP, as the inversion effect shows up in reaction to objects and not to faces.

Results in study 2 replicated the main effects regarding face-inversion, with higher amplitudes in reaction to upright faces than to upright chairs and with higher amplitudes of inverted faces in comparison to upright ones, as well as with a lateralization in the right hemisphere. Neither the right hemispheric-lateralization of OXTR-risk-allele-carriers could be identified, nor longer latencies in reaction to inverted faces in risk-allele carriers of CD38—as found in study 1—could be reproduced.

### Emotional modulation of the Face-Inversion effect

Comparison of happy, angry and neutral upright and inverted faces revealed that N170-amplitudes showed the inversion-effect as well—with the highest negativity in reaction to inverted faces (independent of emotional category), being stronger in the right hemisphere. Concerning effects of distinct emotions, happy faces revealed higher amplitudes than neutral ones, irrespective if upright or inverted. Former results regarding effects of emotional expression on N170 reported no effects of happy emotional expression, but more negative amplitudes in reaction to fearful [2], as well as sad [81] faces.

Results regarding latency revealed longer latencies in reaction to inverted faces, irrespective of facial expression. In contrast to the differences regarding happy faces in amplitude, latency differed in reaction to angry faces compared to happy ones: subjects showed longer latencies in reaction to angry facial expressions than to happy ones. Furthermore, there was an interaction of reaction to angry faces and CD38, so that risk-allele-carriers reacted later to angry inverted faces than the non-risk group. This effect might point to a greater interference in risk-allele carriers in reaction to angry (inverted) faces, whereas this inversion-effect is not evident in reaction to neutral or happy faces. It has been hypothesized that recognition of inverted faces is better in persons with ASD [82, 83]: it is possible that recognition of angry inverted faces is also better in risk-allele carriers of rs3796863 –and therefore, the reaction to angry inverted faces is interfered by the angry expression. As this SNP has also been associated with other social deficits—such as social—phobia [84], interference in reaction to angry inverted faces might, therefore, be possible. Moreover, reaction to upright angry faces was faster in the right hemisphere for the non-risk group of the OXTR-SNP. This effect might indicate that emotion-recognition of angry faces is faster in the non-risk-group than in the risk-group.

One has to state that these effects are only correlational, so nothing can be said about causal effects of the analyzed polymorphisms. Nevertheless, effects of the first study support the hypotheses that CD38 SNP rs3796863 is involved in face-processing similar to that of subjects with ASD or their relatives. Effects of OXTR-gene SNP 53576 rather indicate that the “risk-allele-carriers” process faces as healthy subjects do, whereas G-allele carriers do not show that right-hemispheric lateralization.

The effect of emotional facial expression on N170 amplitude and latency was again observed in the second study: Amplitudes in reaction to upright happy faces were stronger than to neutral ones, indicating that emotional expression indeed has an influence on the N170. Furthermore, latencies were longer in reaction to inverted angry than to inverted happy faces, indicating that, firstly, emotional facial expression influences N170-latency, and that angry inverted faces disturb the processing of faces more readily than happy faces do. Associations of CD38-risk-allele-carriers with later latencies in reaction to inverted angry faces, as well as associations of hemisphere with OXTR could, however, not be reproduced.

### General discussion

Whereas we found molecular-genetic associations of oxytocinergic polymorphisms with reactivity to faces in the N170 in the first study, we failed to do so in the replication sample. What could be replicated, however, are the general effects of face-inversion, independent of oxytocinergic polymorphisms. We investigated the effects of emotional facial expression on N170 on an exploratory basis, as results regarding those effects on the N170 have been heterogeneous [2, 20, 23, 36, 37, 40, 40, 85]. Both studies came to the same result that emotional facial expression directly had an effect on the N170. These effects show that N170 amplitudes-, and latencies are not independent of facial emotional expression, and, therefore, can be influenced by perceiving different facially expressed emotions.

Study 1 yielded significant findings regarding interactions of OXTR and CD38 and face-perception in N170. The replication study, however, failed to do so. It would be too easy to say that results in the first study are clearly due to an Type I error, as it is as well possible that results in the replication study are due to a Type II-error [78]. Although we made sure that the replication study was conducted under the same experimental conditions as the first study, different confounding variables, such as personality, or selective sampling, could have influenced the found results—in both ways, with false-positive findings in the first study, as well as false-negative findings in the second study. This might indicate an incomplete theoretical understanding of the investigated research field, pointing to other possible explanations for the finding, and leading to other research ideas, which is the only way that science can push forward. Another issue which undermines the hypothesis of a Type I error in the first study is the fact that success of reproducibility is correlated with the strength of lower original *p*-values and larger effect sizes in the original study, as well as with the finding that more surprising original findings seem to be less likely to be reproduced than less surprising ones [79]. Surprising effects in this study were for example the right-hemispheric-lateralization effects of OXTR-risk-allele-carriers, because an effect in the opposite direction had been expected. The replication study failed to find any effect of OXTR, however.

The question is, though, if both studies were just underpowered, why are the face-inversion-effects, the lateralization effect, and most importantly, the effects of facial emotional expression consistently found in both the original sample as well as in the replication study? It rather seems to be the case that those effects are the stable, reliable ones, whereas the genotype-interactions could not reliably be found. Those results support the hypothesis that reproducibility is correlated with a lower *p*-value and larger effect size [79].

### Limitations of the studies

The current samples (study 1: N = 72; study 2: N = 78) were comparatively small for molecular-genetic association studies, although, in a comparable range with other combined EEG and molecular genetic studies. Furthermore, subjects were young males—and mainly students—so the results cannot be generalized to all ages or both sexes. However, as subclinical autistic traits are more pronounced in males, they are, therefore, the sample with a higher priority to analyze [86].

The sample of the replication study (N = 78) was only marginally bigger than the first study. Therefore, power-problems occur, as a higher number of subjects would have been necessary in order to maintain the (already small) power of the first study [78, 87]. The procedure of the replication study was the same as in study one, with the exception of another task (emotional Stroop) that subjects additionally conducted in the second study. It is possible that the task influenced the results of the face-inversion-paradigm. However, general face-inversion-effects were replicated from the first study, only the genotype-interactions were not evident in the second study. However, present results rather support the hypothesis that small effect sizes that are close to *p* = .05 (present genotype-interactions in the first study were often close to *p* = .05) are often not possible to replicate, whereas strong effects—such as the general face-inversion-effects (which had strong effect sizes in both studies reported here)–can easily be replicated [79]. This contradicts the hypothesis that the emotional Stroop task interfered with the replication of the results in the second study.

### Conclusion & perspectives

We tried to replicate our findings regarding face-inversion and associations with the oxytocinergic polymorphisms OXTR (rs53576) and CD38 (rs3796863), but could, however, not find these effects in the replication sample. This can be due to many reasons and it does not mean (as already mentioned above) that these effects were definitely accidental. It does, however, show that found effects cannot always be replicated in another study that directly replicates the same paradigm, even in the same laboratory, and with the same stimuli. These results directly correlate with the findings of Nosek and colleagues [79] who criticize the fact that many published results in psychological science cannot be replicated. Present results just emphasize the great necessity to conduct replication studies, especially in the field of psychological neuroscience, in order to come closer to the “true” result. Replications are not a waste of time but an urgent imperative in order to stay a credible and authentic science, which does not only publish effects, independent of the fact if they can be reliably found or not.

This experiment is the first to study individual differences regarding the face-inversion-effect combined with molecular-genetics, and to conduct a direct replication in order to find out if found effects were reliable and stable.

Results are, however, not only highly interesting regarding molecular genetic associations of face-processing, but also regarding general information processing of emotional faces, as an effect of emotional facial expression of the N170 could reliably be found in both the original as well as in the replication sample.

## Supporting Information

S1 Dataset Data regarding N170-amplitudes-/latencies and genotypes of the first study. (SAV)

S2 Dataset Data regarding N170-amplitudes-/latencies and genotypes of the second study. (SAV)
